# Police body-worn cameras and privacy: Views and concerns of officers and citizens

**DOI:** 10.1177/14613557231214383

**Published:** 2023-11-21

**Authors:** Brigitte Poirier, Étienne Charbonneau, Rémi Boivin

**Affiliations:** 6775 École nationale d’administration publique, Canada; 6775 École nationale d’administration publique, Canada; 5622 Université de Montréal, Canada

**Keywords:** Police, body-worn cameras, privacy, work performance, public opinion

## Abstract

Police body-worn cameras (BWC) have been lauded for their potential to increase transparency and accountability by documenting officers’ actions and interactions with citizens. However, despite their widespread use in recent years, several law enforcement agencies have been hesitant to adopt this technology because of privacy concerns. This article explores the views of police officers and citizens from the Canadian province of Quebec towards the use of BWCs. Specifically, it seeks to: (a) understand how officers feel about being monitored by BWCs and (b) assess citizens’ privacy concerns towards police BWCs. A mixed-method research design was used, including interviews and focus groups with 78 police officers, including 46 officers from four pilot sites, and a telephone survey of 1609 residents from the same sites. The results show that officers are concerned about the potential effects of BWCs on their privacy and the privacy of the public. One major area of concern is the impact it may have on their work performance and the use of adaptative measures that support them in carrying out challenging duties. By contrast, most citizens have no reservations about being recorded by a BWC. Certain individual characteristics**—**such as age and perceptions of the police**—**however, were associated with heightened privacy concerns. Without neglecting citizens’ privacy, this study provides insights into the development of BWC policies that preserve officers’ right to privacy and ability to fulfill their duty.

## Introduction

Police body-worn cameras (BWCs) are sometimes thought of as a response to bystanders filming officers. Retired Idaho Police Chief, Scott Haug, shared that, as more of their interactions are filmed by citizens (Bock, 2016), there is “an important opportunity for the officers’ side” to be captured by BWCs (Smith, 2019). However, citizens do not have to record all their whereabouts and interactions and later hand over the footage to their employers. Police officers are increasingly required to do so.

BWCs can be used to monitor the work of police officers, as well as the individuals they encounter or pass by. Although this new form of monitoring has been linked to behavior changes (Lum et al., 2019), little attention has been given to how officers cope under constant surveillance. Officers generally support the implementation of BWCs (Jennings et al., 2014), but some resistance (Young and Ready, 2015) and higher levels of burnout among officers (Adams and Mastracci, 2019) have been reported. The use of BWCs by police officers also impacts citizens who may feel uncomfortable being recorded during personal or sensitive situations (Saulnier et al., 2022).

This article examines the privacy expectations and experiences of police officers and citizens when it comes to the use of BWCs. From qualitative data gathered during a 6-month BWC pilot project, we first explore the attitudes of police officers toward BWCs by examining how they perceive the monitoring aspect of this technology. Using survey data from citizens, we then evaluate their level of concern regarding being filmed by BWCs and whether their opinions vary depending on individual characteristics.

### BWCs to monitor the work of police officers

Police officers are subject to considerable monitoring by their employers. As part of the process of becoming an officer, individuals are typically required to undergo a thorough background check, including verification of their credit history and drug usage, as well as a medical examination (Hickman and Reaves, 2006). Police patrols have also long been tracked using various technologies, including personal radios and Geographic Information Systems (Wain and Ariel, 2014). In addition, new technologies have led to the recording of daily actions for a considerable number of police officers. Along with citizens’ recordings, police departments all over the world have increasingly been purchasing BWCs, with almost half of US police agencies using this technology in 2016 (Hyland, 2018).

BWCs are often touted as tools for increased transparency and accountability (White and Malm, 2020). As a result, they typically receive significant support from the public (Lum et al., 2019). These devices, however, offer a totally new perspective on workers who are traditionally known for their tendency toward closedness. In line with the typology of electronic performance management (EPM) proposed by Ravid and colleagues (2020), the implementation of BWCs can be viewed as a means of evaluating performance or even reducing financial losses (also identified as performance EPM). Although motivations can vary based on location and context, the main goals of BWCs typically are to prevent misconduct by officers and to reduce expenses related to legal proceedings (Löfstrand and Backman, 2021). In the United States, the “control rationale” has been emphasized, particularly in the wake of high-profile cases of police violence against African Americans. By contrast, in European countries, the “protection rationale” is more commonly cited, with BWCs being implemented to protect officers from citizen assaults and false complaints. As outlined by Löfstrand and Backman (2021: 328) the two contrasting rationales behind BWC use—control and protection—“may also have distinctly different consequences for the mental health and well-being of the BWC users”. Indeed, police officers may not be as accepting of the “control rationale” because it may not be as closely aligned with their interests. Also, even though police officers have long been openly observed (e.g., by the public) and generally support the use of BWCs (Jennings et al., 2014; White et al., 2018), these cameras impact them. Earlier research suggests that some officers may exhibit reluctance towards the technology or even experience adverse effects from it. As stated by Stanton (2000: 96), “Employees who are aware of monitoring react to it by forming attitudes and making judgments”. Police officers feel concerned about being watched—and sanctioned—by their supervisors when using BWCs (Makin, 2016; Pelfrey and Keener, 2016). Such fears were expressed before and after the implementation of BWCs (Snyder et al., 2019).

The constant monitoring of workers can also be a significant source of stress (Aiello and Kolb, 1995; Sprigg and Jackson, 2006). In addition to the difficult work–life balance, with officers often working long and irregular hours, the job frequently involves dealing with dangerous or traumatic situations. These conditions induce stress, which has detrimental effects on officers’ mental and physical health (Queirós et al., 2020). Such effects include increased rates of burnout and suicide among officers (Basinska and Dåderman, 2019; Costa et al., 2019). Officers’ stress has also been linked to aggressive behavior during interventions and increased use of force (Kop and Euwema, 2001; Queirós et al., 2013). Monitoring police officers makes a stressful profession more stressful. BWCs were linked to higher rates of burnout among police officers, an effect that could, however, be counteracted by perceived organizational support (Adams and Mastracci, 2019). Even though officers who used BWCs were more likely to experience burnout, their risk decreased when they thought their agency treated them fairly. This finding supports the notion that managing the use of BWCs properly is essential to avoid negative outcomes. It is particularly significant in light of the positive association between the use of BWCs and officer turnover (Schuck and Rabe-Hemp, 2018).

### Citizen privacy in the era of BWCs

The diffusion of BWCs in police departments has led to many debates regarding privacy protection. Whereas advocates of BWCs generally claim that it will enhance police accountability, the recording of officers’ activities was quickly identified as a potential threat to citizens’ privacy. As noted by Blitz (2015: 2), citizens tend to have lower expectations of privacy in public spaces, but it “doesn’t mean, however, that they should expect that every encounter with the police will be part of a permanent video record, accessible to anyone who wishes to obtain it through an open records law, or Freedom of Information Act, request”. The Office of the Privacy Commissioner of Canada (2015: 2) indeed outlined, in its guidance for the use of BWCs by police agencies, that the technology captures what is defined as personal information; that is, “any information which relates to a natural person and allows that person to be identified”. Besides the simple fact of being recorded by a camera, BWCs also have the potential to have advanced features, which can raise concerns about privacy even further. For example, unlike more traditional camera systems, BWCs are increasingly sophisticated and have the potential to be used for facial recognition or capture private conversations due to highly sensitive microphones.

Various segments of the population have been surveyed in empirical research to determine their concerns toward BWCs and privacy. Studies of crime victims’ perceptions of BWCs revealed concerns regarding the recording of their interactions with officers and the management of video footage (Saulnier et al., 2022). As Saulnier and colleagues (2022: 305) note, “Victims’ perceptions of and experiences with BWCs should not be assumed to mirror those of the general public”. When victims are recorded, there is a risk that their experience with the criminal justice system could make them feel victimized again. The police usually are their first point of contact with the system. Therefore, any changes in police practices should be carefully evaluated based on their potential impact on victims.

On the other side of the criminal justice system, suspects and convicted individuals may also be concerned about being recorded by police officers. In their study, Taylor and Lee (2019a) surveyed 907 police detainees across Australia on their views and experiences with BWCs. Although a majority supported the idea of officers wearing BWCs, many who opposed it argued that “the cameras represented an invasion of privacy”. Concerns were mostly related to officers failing to inform citizens when they were being recorded and to the use of cameras in private residences. Some studies also surveyed the general population. According to a study by Crow and colleagues (2017), only a small percentage of residents from two Florida counties were worried about privacy invasion due to BWC use. Specifically, only 11.4% agreed that it would invade residents’ privacy, whereas 7.2% believed it would invade officers’ privacy.

### The present study

According to a survey conducted by Andreescu and Kim (2022) with 2013 US police agencies, almost 40% chose not to purchase BWCs mainly because of concerns about privacy. Hyland (2018) also found that privacy concerns were the most frequent obstacle encountered by agencies using BWCs. As stated by Nissenbaum (2004: 119), “Privacy is one of the most enduring social issues associated with information technology”. The adoption of BWCs by police agencies is not exempt from this problem. Although police activities have been increasingly monitored in recent decades, the use of BWCs now means that all individuals can be recorded.

Despite significant research on this technology, there is still much to uncover about its intended and unintended consequences. The work environment of police officers equipped with BWC, for instance, has received limited attention (Löfstrand and Backman, 2021), and the full impact of BWCs on police officers’ experiences is yet to be fully explored. This is a matter of importance, because the rise in employee monitoring carries the risk of ignoring detrimental psychological effects (Ravid et al., 2022), and police officers already face high levels of stress and burnout (Adams and Mastracci, 2019; Schaible and Six, 2016). The views of those being recorded by police officers are equally important to acknowledge to foster positive community–police relationships. As past studies have shown, privacy concerns remain frequent among diverse segments of the population (Saulnier et al., 2022; Taylor and Lee, 2019b). This study adds to our understanding of how monitoring through BWCs affects the privacy expectations and experiences of both police officers and citizens. It is based on the mixed-method study of a 6-month BWC pilot project in the Canadian province of Quebec.

## Methods

To address privacy concerns related to BWCs fully, we use a convergent mixed-methods design that combines the results of qualitative and quantitative analyses. The two strands of the study interact only when their findings are merged. This design offers the advantage of providing different perspectives on the same problem (Creswell, 2015).

### Qualitative data

The qualitative strand of this study is based on interviews and focus groups with 78 police officers, all employed by the Quebec Provincial Police. Participating police officers were from three distinct groups: BWC users (58.9%), BWC non-users who work alongside BWC users (19.2%), and those who neither use BWCs nor work with BWC users (21.8%). This diverse sample enables the identification of expectations and apprehensions related to BWC monitoring, as well as positive and negative impacts experienced by BWC users. It is also guided by previously defined frameworks that consider the “temporal flow of employee reactions to monitoring” (Stanton, 2000: 88). Although a quick adaptation to monitoring is often anticipated, the long-term effects are less understood. This study provides a more comprehensive understanding of how officers adjust to monitoring by assessing their perceptions at the start and end of a 6-month BWC pilot project. Some 73.1% of the officers were men. On average, participants were 33 years old, and had been police officers for 9 years.

In our individual and group interviews with police officers, we examined their concerns expressed regarding the use of BWCs in monitoring police work, as well as their firsthand experiences with the technology. The interviews (*n* = 83) were held over video conference and lasted approximately 1 hour. Three focus groups were conducted in person and lasted around 2 hours each. Roughly the same interview guide covering the main topics related to BWCs was used for both interviews and focus groups, whether or not officers participated in the BWC pilot. The interviews and focus groups were all held in French. Despite trying our best to capture the essence of the sometime colorful language used by police officers, some nuances could be lost in translation.

### Quantitative data

A total of 1609 citizens were interviewed over the phone; approximately 400 from each of the four sites where BWCs were tested by the Quebec Provincial Police. The survey was carried out by a reputable polling firm. Sampling efforts were made to ensure representative samples from each site in terms of gender, age, and education level (Table 1). Telephone respondents answered 15 questions about their police department's use of BWCs, in both French and English. We report all the results in English; as such, some of the texture can be lost, even if the essence was preserved. The question dealing specifically with privacy in relation to police BWCs is presented in the Results.

**Table 1.** Sociodemographic characteristics of survey respondents.

Characteristic	*n* (%)
Age (years)	
18–34	246 (15.3)
35–54	525 (32.6)
55–74	697 (43.3)
75+	141 (8.7)
Gender	
Female	838 (52.1)
Male	771 (47.9)
Education	
No formal education	174 (10.8)
High school diploma or equivalent	426 (26.5)
Postsecondary diploma, below bachelor’s degree level	683 (42.4)
Bachelor’s degree or above level	322 (20.0)
No answer	4 (0.2)
Household income before taxes ($)	
<25,000	289 (17.9)
25,000 to 74,999	643 (39.9)
>$75,000	520 (32.3)
No answer	159 (9.9)

### Data analyses and integration

Transcripts of interviews and focus groups with police officers were analyzed using Dedoose, a qualitative analysis software. Two researchers coded the transcripts, and any disagreements were resolved in meetings. The material was coded into 12 main themes, including workplace privacy in the face of BWCs. Survey data were analyzed using Stata software. Analyses are presented in the Results.

## Results

### Officers’ views on the impacts of BWCs on privacy

Police officers mostly shared their perspectives on their own privacy (78.1% of coded material), but also discussed the potential impact of BWCs on citizens’ privacy (21.9% of coded material). Table 2 shows sub-theme frequency by category of participant.

**Table 2.** Privacy sub-themes frequency (%), by category of participants in the interviews and focus groups.

	Beginning of pilot (BWC) (*n* = 23)	End of pilot (BWC) (*n* = 45)	Other (no BWC) (n = 15)	Focus groups (no BWC) (n = 3)	Total
Officers’ privacy	33.5	39.5	13.5	13.5	100
Citizens’ privacy	5.9	32.3	26.5	35.3	100

BWC: body-worn camera.

#### Concerns regarding their own privacy

Discussions related to the officers’ privacy revolved around the preservation of personal privacy, the use of recordings by supervisors to evaluate their performance, and the need to keep police tactics from the public. Fears related to privacy expectations included concerns about private conversations with colleagues being recorded. Officers often work in pairs and have long shifts, during which they may discuss personal topics such as family, relationships, or even colleagues. The majority of participants agreed that BWCs should only be activated during interventions, and several emphasized that the cameras should be paused when officers return to their patrol car and have discussions. Some officers reported that during interventions, they sometimes need privacy to reflect and strategize, and they do not want these moments captured on camera. Several officers stressed that they have nothing to hide, but for some, being monitored could add additional stress:

It's not that I feel that there is something that I could be blamed for in my work, but it just seems like […] something that would add more stress, knowing that we are constantly being watched. (Non-user; male officer with 10 years of police experience)

At the end of the BWC pilot, some police officers adjusted to BWC monitoring by informing their colleagues that interventions would be recorded. They explained this was to avoid misinterpretation of their behavior, such as the use of gallows humor, which is a mainstay in policing:

The only influence it really has is [that I warned] my colleagues. Because, in the police, we have to realize it, we tend to turn everything into a joke. It's a bit of therapy. Someone, a civilian, who would follow us from the beginning to the end of the day, would probably be offended by many things that we say, but often, we turn misfortune into ridicule as a form of therapy, you know. (User/end of pilot; male officer with 13 years of police experience)

Others also mentioned that they would initially delay discussions with their partners after the camera was turned off, but over time, they would start to forget to do so. On the 30-second pre-recording feature of BWCs, some participants worried that it could lead to private information being recorded, because any moment could be captured on camera (without audio). These concerns were mostly related to the use of private cell phones, which could lead to passcodes or family pictures (often used as cell phone backgrounds) being recorded. Many participants also reported that the activation button on BWCs was overly sensitive, with some incidents occurring during the pilot. Some of these happened outside interventions, such as during lunch breaks or even in the bathroom, further exacerbating their worries.

Me, personally, I always take off my bulletproof vest [on which BWCs are attached] when I go to the bathroom, so that kind of thing won’t happen to me. But it happened to one of my colleagues, and she wasn’t thrilled. She was like, “Will it be erased?”. Well, no, it stays in the cloud. (User/end of pilot; female officer with 9 years of police experience)

Another cause of concern was the use of recordings for performance evaluation. Views were divided, with some officers accepting that their recordings could be accessed by superiors, whereas others were strongly against it. Most agreed that such evaluations should only be used when necessary and justified and not to nitpick or penalize officers. Not all participants felt that their superiors would feel the need to watch recordings (or have enough free time) without justification, but some remained concerned.

I don’t necessarily want to speak just for myself in my department. I mean, there may be police departments where some superiors could use [recordings] and target some police officers afterward and […] be a little more on their backs, whereas, yes, it's … it should not be the superior who initiates something that ultimately backfires on the officer, simply by viewing the recordings. (Non-user; male officer with 8 years of police experience)

But to say that, hey, they’re going to watch an intervention … on Monday morning, they decide that they’re watching the interventions on the system? I’ve never seen that, and I don’t think they have time to do that with the amount of work there is here, honestly. (Non-user; male officer with 9 years of police experience)

Concerns about performance evaluation were more frequent at the start of the BWC pilot and among officers not using BWCs. These views may have originated from a lack of familiarity with BWCs. However, at the end of the pilot, participants related events that negatively impacted their trust in management. In one participating department, for instance, the officers who took part in the pilot were informed that some of their colleagues had viewed a recorded intervention that they were not supposed to see. The dissatisfaction among the officers was further amplified by the fact that even those who were involved in the intervention had not watched their recordings. A common complaint was that officers had to go through a complex procedure to view their own footage, whereas citizens who received a road traffic ticket could easily access recordings through a link provided by the agency.

Some officers also pointed out the need to protect police tactics. These concerns were more frequent by the end of the BWC pilot, because participating officers had faced situations in which recording had the potential to jeopardize the confidentiality of police strategies, including meetings with informants and containment techniques for when a crime is in progress.

I’m someone who likes working narcotics, with a colleague […] during the 6 months that we had the camera, it is certain that we had fewer people with whom we had to deal, who opened up on information, either on narcotics or any criminal information. Because the camera was on, for example, and people, they don’t want their names to come out. (User/end of pilot; male officer with 8 years of police experience)

#### The impacts of BWCs on citizens’ privacy

During the interviews and focus groups, officers also expressed concerns about citizens’ privacy, acknowledging that not every situation should be documented. Interventions with vulnerable individuals, such as victims, children, and those with health issues, were identified as potentially problematic to record. As some pointed out, interventions where there is no criminal concern may not need to be recorded. Some participants expressed that “bad moments” in one's life should be kept private, whereas others recognized the importance of blurring the faces of bystanders. An officer compared his concerns with the duty of confidentiality for healthcare professionals, adding that any event that is not likely to be discussed in court should not be recorded or made public. For example, several participants explained that events unrelated to police work, such as car accidents resulting in death, should not be recorded, including the announcement to families. An officer who participated in the pilot explained that despite the activation policy being clear, their personal feeling of empathy would make them question whether it was necessary to record:

I mean, a person who's, like, unclothed, naked, are you gonna start … am I comfortable filming it, and all that? So it's more at this level that, sometimes, I had reservations. A person who is bedridden, who requires care, and all that, are we going to start filming [them] naked, and all that, or the paramedics who take their vital signs, and all that? […]. We agree, of course, that these images will never be reproduced and that no one will allow these images to be viewed. But it was more at the level of the values… of my values, is it something that I want to do? (User/end of pilot; male officer with 6 years of police experience)

In certain cases, however, it may be appropriate to document sensitive situations. At the beginning of the pilot, a complaint was filed about officers recording in a local hospital. A decision was quickly made to restrict the officers from using their BWC while inside the hospital. Officers explained that they sometimes recorded agitated or violent individuals. Although they acknowledged the decision to restrict recording to protect the privacy of the hospital patients, some expressed their disappointment:

We also wanted to demonstrate that we continue to assist [the hospital workers]. There are times, in fact, we are also called to assist them even inside the hospital. Because the person is not controlled […] is too aggressive for them to handle. So, we must continue to intervene by using force. And if we can’t record and after that, they tell us that the intervention was not appropriate, well, we didn’t have the chance to record. (User/end of pilot; female officer with 3 years of police experience)

When discussing interventions with domestic violence or sexual assault victims, several participants also suggested that recording should not be their priority. Recording vulnerable people may impact the helping relationship they are trying to establish because of the potential impact on personal privacy. Thus, there would be no reason to record such events:

Maybe, in the end, if we didn’t have the cameras, we would have had her whole statement, and we would have had more details. Then, with the camera, well then, she may have closed herself off, and then she omitted to say certain details. And then, I think we are losers in this. (User/beginning of pilot; male officer with 5 years of police experience)

Another officer highlighted the importance of considering the severity of the crime, because it could have a different impact on the willingness of victims to record their interactions with the officers. Some officers also felt that citizens might not agree with being recorded in their own homes. An officer using BWCs explained that people might have different reactions when they are being recorded, with some “who don’t have much ground for self-reproach” and others often asking the camera to be turned off:

There are those who tell you: “Don’t show this to anyone”. You reassure them, you tell them that we won’t show it, and … I never turned it off because, anyway, we were told not to turn it off […] but sometimes I had to reassure the person by telling them not to worry about it, that it was to protect the—us and them—anyway, you understand—the magic phrase that we were told to say, that it's to protect everyone. (User/end of pilot; male officer with 11 years of police experience)

According to another officer, some individuals may not have the immediate instinct to advocate for their privacy:

It's still an environment, I would tell you, you know, there are a lot of social assistance recipients, people that … you know, mental health, substance use issues. So, often, I think these people don’t have the same reflex to say, “Hey, it's my private life, at home”, or whatever. Because that's not the kind of … lingo that they often use, it's more “OK. This is happening. Come home”. (User/beginning of pilot; male officer with 4 years of police experience)

For some participants, this risk to citizens’ privacy should be considered in the overall context of protection. As explained by a male officer, any event posing a risk to mental or physical safety should be recorded, regardless of location, and any individual who causes such risk should be prepared to be filmed. Another participant argued that BWCs are used to document the work of officers, and therefore there must be only limited circumstances where recording is not permitted:

We are filmed from A to Z by citizens. But we too shouldn’t have any restrictions to film whatever we want. In the end, that's what cameras are for, to see what we’re doing. If we cut a good part of it, it will raise other questions. I think we should film everything and, at worst, redact. But with certain reservations, sometimes for intimacy, then confidentiality, care should be taken. (Non-user; male officer with 5 years of police experience)

Several officers thought that despite the privacy concerns, there should be no restriction to recording, with some suggesting that rather than restricting when or where they can record, they should rely on redaction techniques to protect privacy when necessary. For some participants, it appeared necessary that citizens realize that they may be recorded when interacting with police officers and thus should be aware of the risks to their private life.

### Results from the citizens’ survey

As part of our survey of residents in the four pilot sites, we included an open-ended question regarding their concerns about being filmed by a police BWC. There were three versions of that yes/no question. One-third of respondents (control) were asked: “Do you have any fears or reservations about being filmed (yourself) by a police officer's hand-held camera?”. The second (treatment 1, or T1) and last third of respondents (treatment 2, or T2) were respectively asked about “citizens in your area” and “citizens of Quebec”. being filmed. Respondents who answered “yes” to having reservations were invited to explain their reasons in their own words.

**Figure 1.**
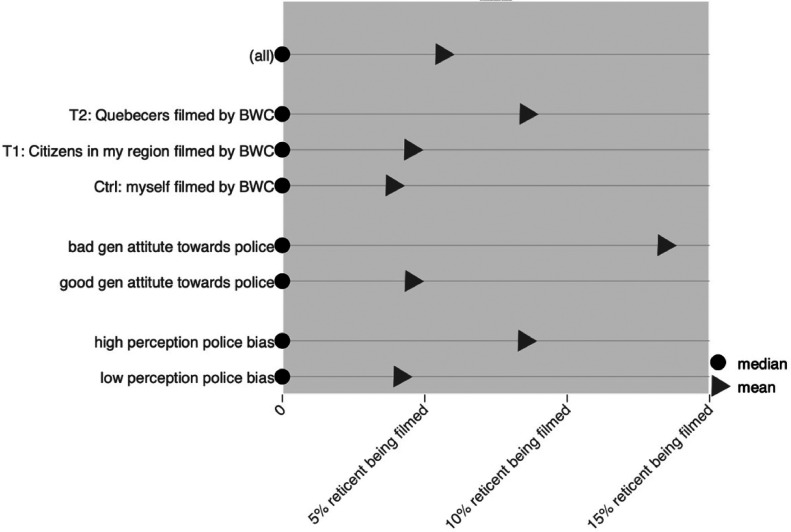
Proportions of respondents expressing fears or reservations about being filmed by a body-worn camera.

Globally, of the 1609 respondents, only 5.6% answered positively about having reservations and fears of being recorded. Given the limited occurrence, it is risky to parse out these respondents along sociodemographic dimensions. The respondents fearing and having reservations about being filmed tended to be older than the large majority who did not (*z* = 2.17; *p* < .05). The profile of reticent respondents did not vary by income, gender, or ethnic origin. However, variations were observed along the two Perceptions of Police Scale (POPS) dimensions (Nadal et al., 2005): general attitude towards the police and perceived presence of bias from the police. Respondents with a good general attitude towards the police (*z* = −3.26; *p* < .001) and those who did not see the police as biased (*z* = −1.69; *p* < .01) were less likely to be part of the reticent respondents. There were some differences among the proportion of reticent respondents depending on the experimental arm. Whereas 3.9% of respondents expressed fears or reservations when they themselves were being filmed (control), the proportion went up to 4.5% and 8.5% when the question mentioned that citizens of their region (T1) or citizens of their province (T2) were going to be filmed. The overall pattern is statistically significant (χ^2^ = 13.01; *p* < .001). That difference comes from the differences between the T1 and T2 (*t* = −3.25; *p* < .001), and the control group and T2 (*t* = −2.63; *p* < .05). Figure 1 shows differences in the proportion of respondents expressing fears or reservations about being filmed, along with experimental variations in the question and the two POPS dimensions.

The fears and reservations of respondents, based on their written comments, are summarized in Table 3. As mentioned above, respondents who were assigned to T2 were twice as likely to express reservations compared with those assigned to the control group or T1. The most common reasons for concerns were related to officers’ misuse of BWCs and the lack of privacy. Some respondents expressed concern about the possibility of citizens or children being recorded without their consent. Others were worried that officers may forget to activate their cameras or start recording during situations that do not necessitate it. There were some differences between the three experimental arms, with respondents in the control group being more likely to report concerns about the potential negative effects of using BWCs on citizens, including the possibility of misunderstandings arising from the footage.

**Table 3.** Categorization of themes from the answers to the open-ended question by experimental arm.

Reasons for fears or reservations^a^	Control (“myself” filmed)	T1 (“citizens of my region” being filmed)	T2 (“citizens in Quebec” being filmed)	Total
Lack of privacy (filming individuals without consent, filming children, etc.)	43.8%	33.3%	46.7%	42.2%
Misuse by police officers (failure to activate, filming outside necessary situations, lack of supervision, etc.)	31.3%	50.0%	43.3%	42.2%
General disapproval of BWCs (seen as an excessive measure, lack of information on BWCs, dislike BWCs, etc.)	12.5%	11.1%	6.7%	9.4%
Possible consequences on citizens (misunderstanding of footage, incomplete footage, repercussions for citizens, etc.)	12.5%	5.6%	3.3%	6.3%
Total	100%	100%	100%	100%

^a^ We removed responses from those who either did not know or chose not to answer.

BWC: body-worn camera.

## Discussion

Police officers and civilians are subjected to BWC surveillance in different settings: officers are filmed in their work environment, whereas civilians may come across BWCs in both private and public spaces. Both groups, however, have concerns about their privacy being compromised by BWCs. The following discussion examines how the inclusion of a new witness in their interactions is perceived by these actors and how our findings can inform BWC policies.

### Privacy, relations, and job performance

Police officers are generally in favor of BWCs (Lum et al., 2019). Nevertheless, some may still feel discomfort with the idea of being under constant surveillance. As highlighted by this study, officers need to adjust to the use of cameras in their daily activities, with one area of concern being how they interact with each other. Officers are not particularly worried about getting caught engaging in inappropriate behavior. However, given the sometimes difficult nature of their job, they develop various coping mechanisms, including the use of humor (Pogrebin and Poole, 1988), which helps to create a bond among colleagues (Gayadeen and Phillips, 2016). In addition, group solidarity is an important element of police culture that can be observed in the conversations officers have with each other. As explained by Campeau (2015: 670), “police culture is used by officers as a resource to make sense of their occupation lives”. Our findings suggest that officers can be apprehensive about the idea of exposing these adaptations (or lifting the “blue curtain”). Whereas several were concerned about their tactics being revealed to the public, others also feared that showing their personal interactions may lead to negative consequences or portray them in a negative light. Pelfrey and Keener (2016) also found that officers may be apprehensive about potential disciplinary action for using “strong language” or raising their voices during interventions. Previous research has shown that using BWCs may have adverse effects on the mental health of officers (Adams and Mastracci, 2019). It is also possible that their apprehension about privacy limits their capacity to rely on mechanisms to cope with their job. It is essential to examine the long-term consequences of these concerns on their well-being.

When it comes to police culture, trust is also an important factor. Many officers expressed that they have faith in their superiors not to misuse BWC recordings to keep an eye on and punish them. Once trust is lost, however, it can be difficult to regain. Our findings emphasize the importance for organizations to establish mechanisms that promote trust between officers and their superiors, including controlled access to footage and proper video redaction techniques. According to White and colleagues (2017), most agencies allow supervisors to review BWC footage in instances in which officers have used force or when a complaint has been filed, as well as for performance evaluations. Our study suggests that officers can get used to the idea: by the end of the pilot, performance evaluation was less of a concern. This is consistent with other studies that showed officers had a more positive attitude toward BWCs after using the technology (Gaub et al., 2016).

### Balancing efficacy and citizens’ privacy

Our study also examined perceptions about the potential privacy threats of BWCs to citizens. First, we assessed whether citizens had concerns about being recorded by BWCs, and whether their personal characteristics were correlated to their privacy beliefs. Our findings align with those of previous studies (Crow et al., 2017; Grossmith et al., 2015) and show that only a small minority (<6%) of citizens are worried about privacy issues related to BWCs. Indeed, although privacy rights groups have been voicing concerns about BWCs (White and Malm, 2020), it seems that most citizens are accepting of being recorded. Our sample, however, was drawn from the general population, which limits our ability to examine certain underrepresented groups, like crime victims, who are at a higher risk of having sensitive information recorded (Saulnier et al., 2022). Nonetheless, we did identify some factors that contribute to heightened levels of apprehension among the public, such as age and perceptions of the police. We found that older respondents were more likely to have reservations about being recorded, which could be attributed to a stronger desire for privacy protection (Quan-Haase and Ho, 2020). As expected, respondents who were more favorable toward the police had fewer concerns about being recorded. Interestingly, privacy concerns were more prominent when it was mentioned that Quebec citizens would be recorded, in contrast to respondents themselves being monitored. This finding is consistent with those of the Edmonton Police Service (2015), which suggests that the general public has higher privacy concerns than those who directly interact with the police.

Our study also helps us gain a better understanding of how officers, who are at the forefront of BWC usage, perceive the impact of this technology on citizens’ privacy. Officers had many reservations about filming citizens, especially if the recording served no purpose (e.g., could not be used as evidence). The officers provided several reasons for their concerns, including the possibility that citizens may become less willing to engage in discussions with them. These types of concerns are not uncommon (Edmonton Police Service, 2015). However, despite officers’ good intentions, the completion of their police duty remains a priority. As White and Malm (2020: 51) wrote, “Every benefit can have a cost; and in the case of BWCs, the cost of increased transparency is decreased privacy”. BWCs were introduced in police organizations during a period of legitimacy crisis, and officers are concerned about being falsely accused of misconduct. It should not come as a surprise that those who have the power to activate the cameras would prioritize their own interests when they feel they are threatened.

### Limitations and future research

Our findings based on interviews and focus groups with police officers were limited to instances of work and citizen privacy. The concerns they expressed were in response to questions on a range of subjects, rather than targeted questions on privacy. Hence, they represent the organic preoccupations of police officers in many facets of their work. However, by not prodding all respondents specifically on niche aspects of privacy, some rare yet real risks might have been overlooked. In addition, the police agency under study does not cover major cities where respondents wary of or even hostile to police officers are present. As we have shown, trust in the police explains more variations in privacy views than sociodemographic dimensions. As such, our large sample of citizens should be more trusting than their urbanite counterparts. We are unsure whether this reservoir of trust in the police biases the positions one way or another. Even if there is a small but vocal proportion of citizens who are hostile to the police, they may still want BWCs despite the privacy cost. Finally, one major limitation of our study is that we did not question citizens about the concerns they may have had regarding the work privacy of police officers. This presents a promising area for future researchers to explore.

## Conclusion

According to Nissenbaum (2004), privacy is more complex than the dichotomy of public and private spheres. In her view, privacy is violated when information norms—that is, appropriateness (what is considered suitable to disclose in a given context) and distribution (how information is shared among parties involved)—have been transgressed. The use of BWCs by police officers has brought new visibility to policing. It is usually anticipated that, by capturing interactions between citizens and officers, the community–police relationship will be improved. Although good outcomes are possible, there are also negative ones to consider, and threats to privacy have been a major concern since the initial implementation of this technology. The concerns revolve around both the appropriateness of recording and who should be allowed to access the footage.

Ensuring the privacy of citizens remains a major consideration that should not be overlooked. Although it may not have been at the forefront of officers’ minds at the beginning of the BWC pilot, it often became increasingly important towards the end of the 6-month period. Interestingly, there appeared to be minimal concerns from citizens regarding the privacy costs of surveilling police officers with BWCs. This suggests that BWC policies may be better to prioritize officers’ reservations rather than the public's (which are relatively minor). Our research provides valuable insight for the development of BWC policies that consider the potential impact of the technology on officers’ performance and overall well-being. In an era in which police recruitment and retention are difficult in Canada (Rigaux and Cunningham, 2021) and elsewhere, BWC policies should not make the workdays of police officers impossible by taking away their work privacy. Police officers are not only responsible for enforcing laws, but they are also workers who deserve to be treated with respect and dignity.
